# Books Reviewed

**Published:** 1868-08

**Authors:** 


### Books Reviewed.

Circular No. 1, War Department, Surgeon-General's Office, Washington, June 10, 1868. Report on Epidemic Cholera and Yellow Fever in the United States Army during 1867. By Brevet Lieut. Col. J. J. Woodard, Ass't Surgeon U. S. Army.

This is a statistical report embracing a wide field and containing numerous well attested facts. The value of the contributions of the Surgeon-General's Office to medical literature is inestimable, and can only now be imagined, since it must require years to digest and fully appropriate the vast amount of fact here opened up for the future study of the profession.

A Medical Report upon the Uniform and Clothing of the Soldiers of the U. S. Army.

This report is made by Alfred A. Woodhall, Assistant Surgeon and Brevet Lieut. Col. U. S. Army, upon the following order issued to medical directors in August, 1867:

"Sir:—You are respectfully requested to call upon medical officers of experience serving under your command, for their opinions regarding the hygienic fitness of the present uniform and allowance of clothing for enlisted men, and to invite suggestions for their modification."

The report is based upon the reports of more than one hundred and twenty professional men, representing nearly as many stations, and is full of valuable suggestion. The author remarks in conclusion:

"The perusal of the various *Statements* appended to this Report will show the interest and intelligence with which the medical officers have discussed the subject. There will be found described errors and horrors belonging to the existing uniform that could be barely touched upon in the foregoing pages, and fertile suggestions for their relief that were equally lightly passed over. Underlying the whole is the conviction that while our army is surpassed by none in parts of its equipment and in the wonderful achievements it has accomplished, there will be open to it the avenue of still greater efficiency and success when its daily government shall be guided by the teachings of rational preventive medicine and hygiene."

"Position" in the treatment of Chloroform Poisoning.—By E. L. Holmes, M. D.

Dr. Holmes says:—"Whatever may be the obscure causes of fatal results from the use of chloroform, I believe the danger, in by far the larger proportion of cases, depends upon a tendency to death by syncope. To overcome this tendency, it is necessary to stimulate the nervous centres. This may be done by causiug a column of blood to press upon the vessels of the brain. It is not sufficient to remove the pillow from the head and place it under the hips. It is necessary that the whole body be placed upon a steep, inelined plane, to force as much blood as possible, by gravitation, into the brain. I believe this is of more importance than any of the methods usually described by writers on the subject. It should take precedence to the withdrawal of the tongue, artificial respiration, galvanism, or stimulants. This remedy can always be applied without delay, and can be followed by any others which may seem desirable."

